# Examining trends in family planning among harder-to-reach women in Senegal 1992–2014

**DOI:** 10.1038/srep41006

**Published:** 2017-01-20

**Authors:** Francesca L. Cavallaro, Lenka Benova, David Macleod, Adama Faye, Caroline A. Lynch

**Affiliations:** 1 Department of Infectious Disease Epidemiology, London School of Hygiene & Tropical Medicine, London, UK; 2 Institut Santé et Développement, Université Cheikh Anta Diop, Dakar, Senegal

## Abstract

Recent increases in family planning (FP) use have been reported among women of reproductive age in union (WRAU) in Senegal. However, trends have not been monitored among harder-to-reach groups (including adolescents, unmarried and rural poor women), key to understanding whether FP progress is equitable. We combined data from six Demographic and Health Surveys conducted in Senegal between 1992/93 and 2014. We examined FP trends over time among WRAU and subgroups, and trends in knowledge of FP and intention to use among women with unmet need for FP. Our results show that percent demand satisfied is lower among rural poor women and adolescents than WRAU, although higher among unmarried women. Marked recent increases have been observed in all subgroups, however fewer than 50% of women in need of FP use modern contraception in Senegal. Knowledge of FP has risen steadily among women with unmet need; however, intention to use FP has remained stable at around 40% since 2005 for all groups except unmarried women (75% of whom intend to use). Significant progress in meeting the need for FP has been achieved in Senegal, but more needs to be done particularly to improve acceptability of FP, and to strategically target interventions toward adolescents and rural poor women.

The fertility decline observed in low- and middle-income countries over the last four decades has been less pronounced in sub-Saharan Africa (SSA) where total fertility remains at 5.1 children per woman, compared to 2.7 in other world regions^1^. Low contraceptive uptake contributes to this sustained high fertility: the modern contraceptive prevalence rate (MCPR) in SSA is less than half the rate in other regions (24% versus 53% in Southeast Asia and 81% in East Asia)^2^, and the unmet need for family planning (FP) is higher^3^. Within SSA, the MCPR was lower in West Africa than East and Southern Africa^4^.

In Senegal, advertisement and distribution of contraceptives was prohibited until 1980, when the 1920 French anti-contraception law was repealed^5^. The first private FP clinic opened in Dakar in the early 1960s^6^, followed by other localised projects, and a national programme was introduced in 1990 with the aim of coordinating existing FP services. Subsequent increases in contraceptive use were slow, and one-third of married women still had an unmet need for FP in 2010–11 7. In response, the Senegalese Ministry of Health launched an ambitious National Family Planning Action Plan in 2012, announcing a national target of 27% MCPR by 2015 among women of reproductive age in union (WRAU) – from 11% MCPR in 2011 – through increased availability of FP services and commodities, as well as a broad communication and advocacy campaign^8^. Although adolescents and rural women are mentioned as priority groups in the Action Plan, no targets were set among these groups.

Within SSA, large disparities in FP use exist between and within countries, resulting from a number of barriers to contraceptive uptake, including lack of knowledge and acceptability of contraception, poor geographical and financial access to FP, and poor quality of FP services^9^,^10^,^11^,^12^. Different groups of women face different obstacles to FP use; in this analysis we focus on three groups with greater (albeit different) barriers to access, considered “harder-to-reach” groups: adolescents, unmarried and rural poor women. Rural and poorer women face greater geographical and financial barriers to FP, resulting in large urban/rural and wealth gaps in contraceptive use and unmet need that continue to be observed globally^13^,^14^ including in Senegal^15^.

Adolescents and unmarried women may face other challenges in accessing FP services, in addition to potential geographical and financial restrictions. In Senegal, strong norms exist against premarital sex, and although there are no legal restrictions to providing FP to young or single women, providers often promote abstinence until marriage^16^. A simulated client survey conducted in 1995 found that none of the six clients aged 15–24 who requested contraceptives received them^17^. In one recent study of urban health facilities, around half of public providers reported setting minimum age restrictions for pills and injectables (two of the most popular methods among young women), while at least 12% reported not providing contraception to unmarried women for these methods^6^. Few other studies have looked at unmarried women, but in 2001, only 37% of single sexually active women aged 15–24 reported using contraception in 18 SSA countries^18^.

Senegal is a key hub in West Africa, a region with very high fertility^19^. A rapid rise in contraceptive use among WRAU has been reported recently, the MCPR almost doubling from 12% in 2010–11 to 20% in 2014^7^,^15^. However, attention is rarely given to trends over time among harder-to-reach groups such as young or single women, with a few exceptions^18^,^20^. Furthermore, reporting MCPRs is of limited value when comparing different groups of women considering the need for FP is likely to vary between them, and the percent demand satisfied has been proposed as a more useful indicator^21^.

While the national target of 27% measures FP progress among all WRAU in Senegal, the aim of this study was to determine whether substantial advances in meeting the need for FP also occurred among specific harder-to-reach groups (namely adolescents, unmarried women, and rural poor women), by comparing trends in three key FP indicators over time: MCPR, unmet need for FP, and percent demand satisfied. Our second objective was to examine knowledge of FP and intention to use FP among women with unmet need in order to better understand obstacles to contraceptive uptake among these groups.

## Methods

### Study setting

Senegal is a lower-middle income country of 15 million people in West Africa^22^. Most of the population is concentrated in the west and centre of the country, with over 40% of the population located in the peninsula regions of Dakar and Thiès^15^. Population density is much lower in the north and south-east. The health system in Senegal follows a hierarchical model, with each of the 14 regional medical offices responsible for overseeing health districts in the region. Health districts typically include one health centre and a network of rural health posts, some of which oversee affiliated health huts^23^. Health huts are community-level facilities, usually staffed by a community health agent paid by village health committees. National reproductive health guidelines indicate that all modern FP methods should be provided at all levels of the health system, with the exception of IUDs and diaphragms (all facilities excluding health huts) and sterilisation (in surgically-equipped health centres and hospitals only)^24^.

### Data source

All data used in this analysis come from Demographic and Health Surveys (DHS)^25^, standardised and nationally representative surveys of women of reproductive age collecting information on fertility and reproductive health. These surveys are publicly available on the DHS website (www.dhsprogram.com). We compiled six DHSs conducted in Senegal (1992/93, 1997, 2005, 2010/11, 2012/13, and 2014) in a single database. The 1999 survey was excluded because data collected did not follow the standard DHS model. All variables included in the compiled dataset were checked and recoded to ensure standardised response categories across surveys. Although DHS questionnaires are standardised, changes may be made to questions or response codes in successive surveys (for example, the response codes for individual contraceptive methods currently used is different in different rounds).

### Definition of key variables

#### Modern contraceptive methods and unmet need

Pills, injectables, implants, intra-uterine devices (IUDs), male and female condoms, male and female sterilisation, diaphragms, spermicides, and sponges were categorised as modern contraceptive methods. The revised DHS definition of unmet need for family planning was used^26^: women not wanting another birth in the next two years and not using modern contraception, or whose last pregnancy was not wanted, were considered to have an unmet need for FP. This definition of unmet need assumes that all married women are sexually active. Unmarried women are considered to be exposed to conception only if they report sexual activity in the past month; those reporting no sexual activity in the past month were considered not to have a need for FP.

The 0.4% of women with missing information on unmet need were considered not to have an unmet need. There were no missing data for contraceptive use.

#### Easier- and Harder-to-Reach groups

**Figure 1:**
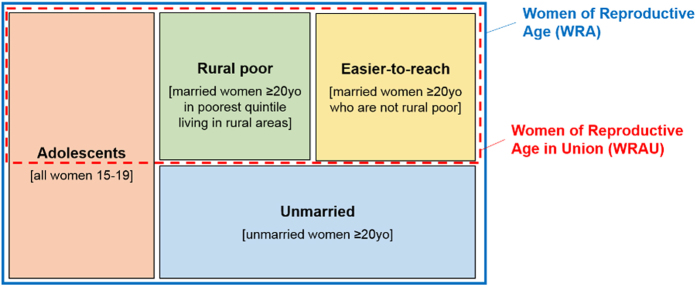
Definition of easier-to-reach and harder-to-reach groups. Note: the size of different boxes does not represent the proportion of each group in the Senegalese population. Adolescents, unmarried women and rural poor women are considered harder-to-reach groups.

We defined three mutually exclusive harder-to-reach groups (Fig. 1): adolescents (aged 15–19), unmarried women (never-married and separated/widowed women aged over 20), and rural poor women (married women aged 20 and older living in rural areas whose household wealth score fell in the poorest quintile). These three proposed harder-to-reach groups were selected because geographical and financial barriers, and provider restrictions emerged from the literature as the biggest obstacles to FP use in Senegal. All women not classified into one of the three harder-to-reach groups were considered easier-to-reach women (married women aged over 20 who are not rural poor).

Based on the definitions used in this study, all easier-to-reach and rural poor women are married, while none of the unmarried women are. In contrast, the adolescent group includes some married and some unmarried women. Therefore, the WRAU group among whom the national target was set includes all easier-to-reach, all rural poor, and some adolescent women (Fig. 1). Wealth quintiles were not calculated before the 1997 DHS, thus rural poor women cannot be identified for the first survey: accordingly, trends for easier-to-reach and rural poor women are reported from 1997 onwards.

#### Obstacles to contraceptive use

We examined obstacles to contraceptive use within Coale’s framework of fertility decline^27^, as revisited by Lesthaeghe and Vanderhoeft^28^. According to this framework, three preconditions are necessary for couples to adopt contraceptive use: *readiness*, or a clear micro-economic benefit of birth control; *willingness*, or acceptability of practicing contraception; and *ability*, or knowledge of and access to contraceptive methods.

By definition, women classified as having an unmet need want to postpone births, and are therefore *ready* to use FP within this framework. Our objective was to examine *willingness* and *ability* among women with unmet need, using an approach adapted from Machiyama and Cleland^29^. Our proxy measure for *willingness* was women reporting they intend to use contraception in future, and for *ability*, women knowing of both pills and injectables (the two most popular contraceptive methods in Senegal) and of an FP source.

### Statistical analyses

We first described the socio-demographic characteristics of women in each group of interest. Trends in FP use and coverage were compared between WRAU – among whom the national MCPR target was set – and each of the groups of interest by calculating the MCPR (percentage of modern contraceptive users among all women), unmet need for FP (percentage of women with unmet need among all women), and percent demand satisfied (percentage of modern users among women in need of FP) in each group and survey year. The absolute yearly rates of change for each indicator were calculated for each interval between surveys by dividing the absolute change between surveys by the number of years between surveys.

We examined proxies of ability and willingness to use FP in the groups of interest by calculating the percentage of women with unmet need who had knowledge of, and intention to use FP, over time. Determinants of knowledge and intention among all women with unmet need (including parity, rural/urban location and education) were explored using bivariate and multivariable logistic regression models. All socio-demographic characteristics were included in the multivariable models.

All analyses were conducted in Stata SE software (version 14; StataCorp) and took into account sampling weights, as well as clustering and stratification where appropriate.

### Ethical approval

The DHS obtain ethical approval in the host country and follow ethical practices including informed consent and voluntary participation of respondents and ensuring privacy and confidentiality in data collection and processing^30^. Our secondary analysis was approved by the Research Ethics Committee of the London School of Hygiene & Tropical Medicine.

## Results

The total sample used for analysis included 62,317 women aged 15–49 across the six surveys. Table 1 presents the socio-demographic characteristics of women in each group in the most recent survey. In 2014, almost half of easier-to-reach women had 2–4 children, one-third were in polygamous marriages, and 58% lived in urban areas, principally in the west of Senegal. More than half of easier-to-reach women (58%) had received no formal education. Among adolescents, one quarter were married and 14% had started childbearing. Around half of 15–19 year olds lived in urban areas, and 44% had reached secondary education or above. Most (71%) of women in the unmarried group had never been married, while 29% were divorced, separated or widowed, and two-thirds had at least one living child. Unmarried women tended to be more urban, more educated, and from richer households than other groups. Rural poor women had higher parity than other women, with 40% having 5 or more children, and most frequently resided in central and southern Senegal, in contrast to the other groups concentrated in the west. Almost 90% of rural poor women had received no education.

### Modern Contraceptive Prevalence Rate (MCPR)

**Figure 2:**
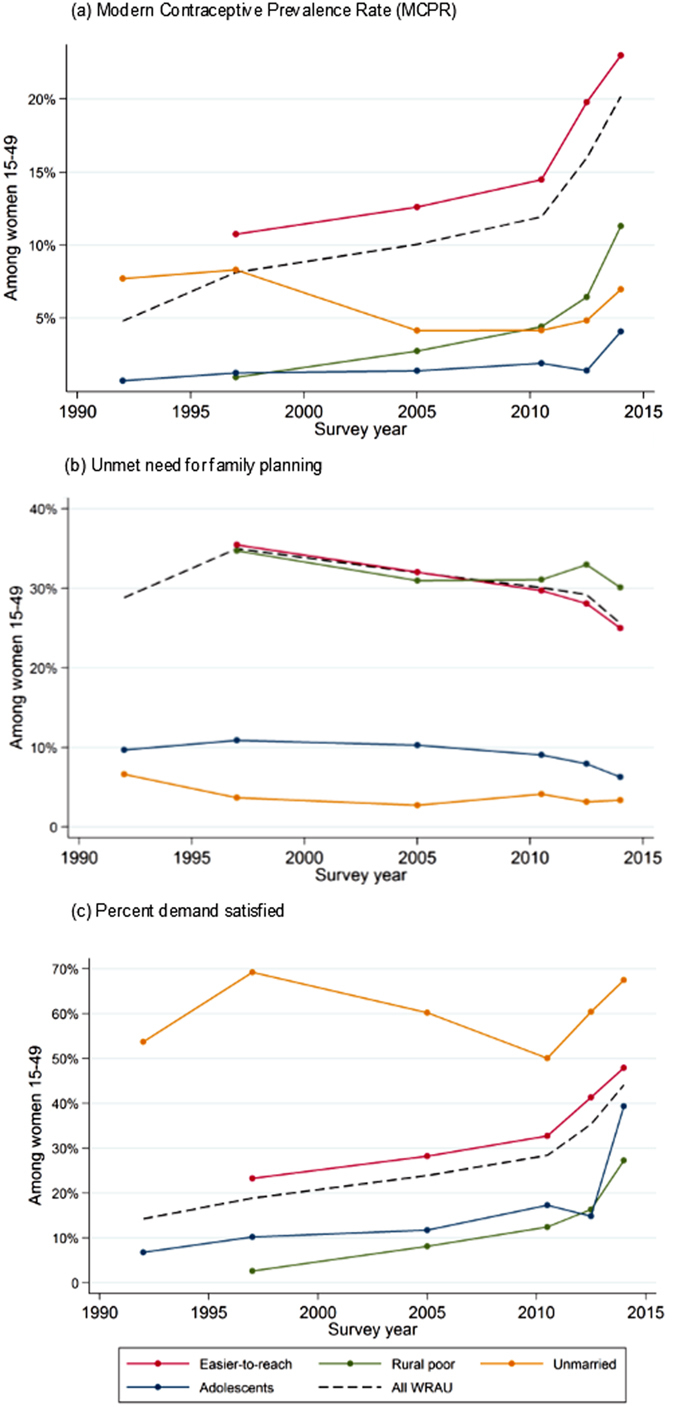
MCPR, unmet need for family planning and percent demand satisfied among all women of reproductive age in union (WRAU), and easier-to-reach and harder-to-reach groups.

The MCPR rose steadily among WRAU from 4.8% in 1992/93 to 11.9% in 2011, after which the pace of increase accelerated, to 20.2% by 2014 showing rapid progress toward the 27% national target (Fig. 2a). The average absolute increase was at most 0.8 percentage points per year prior to 2010–11, compared with above 2.1 percentage points per year thereafter (Supplementary material 1). Easier-to-reach women displayed similar trends to WRAU, but at slightly higher contraceptive prevalence levels. Among harder-to-reach groups, the recent increase in MCPR was more pronounced among rural poor women, gaining 3.5 percentage points per year between the last two surveys, and less so among adolescents and unmarried women, who gained less than 2 percentage points.

### Unmet need for family planning

The unmet need for FP remained relatively stable among all groups over the study period (Fig. 2b). Among WRAU, unmet need rose from 29% in 1992 to 35% in 1997, before decreasing to 26% in 2014. This decline accelerated between 2012–13 and 2014, later than the accelerated increase in MCPR. These trends are closely mirrored among easier-to-reach women.

Rural poor women had similar levels of unmet need to WRAU, although it has fluctuated around 30–33% from 2005 onward while WRAU have experienced a continuous slow decline since 1997. In contrast, fewer adolescents and unmarried women were categorised as having an unmet need for FP (around 6% and 3% in 2014, respectively), and a slight decline occurred since 2010–11 among adolescents, though not among unmarried women.

### Percent demand satisfied

The percent of demand for FP satisfied by modern contraceptives follows similar trends to contraceptive use among WRAU and easier-to-reach women – a slow rise until 2010/11, followed by a rapid increase (Fig. 2c). The percent demand satisfied reached 44% among WRAU and 48% among easier-to-reach women in 2014. The percent demand satisfied was highest among unmarried women, fluctuating around 60% since 1992. Rural poor women had the lowest percent demand satisfied across groups, reaching only 27% in 2014, while adolescents experienced a rapid increase from 15% in 2012/13 to 39% in 2014.

### Ability and willingness to use FP among women with unmet need

**Figure 3:**
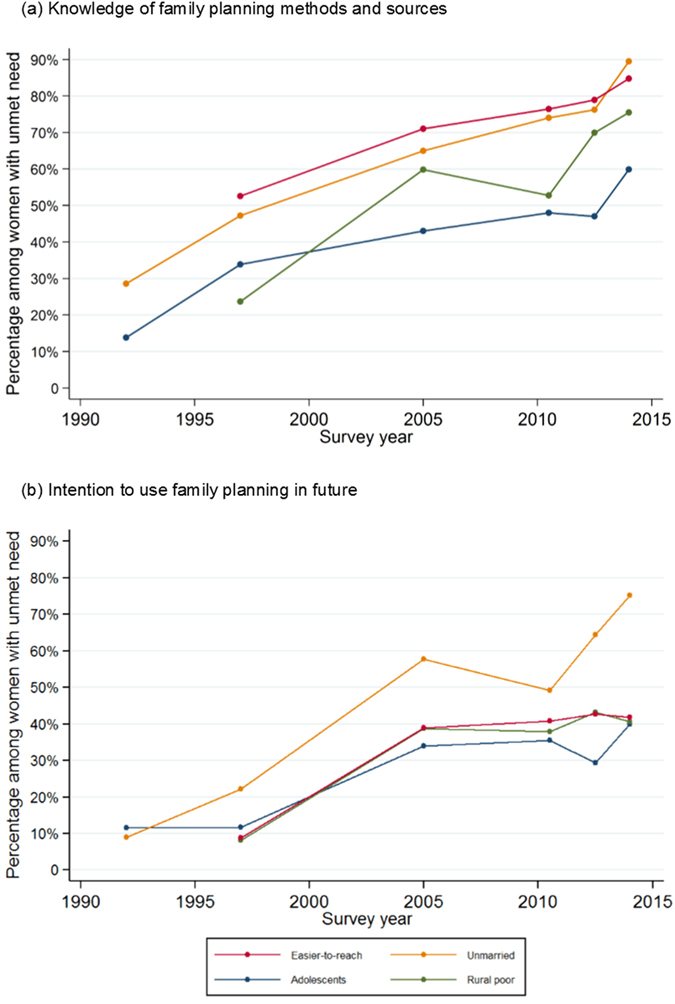
Percentage of women with unmet need who had knowledge of and intention to use family planning over time in Senegal, among easier-to-reach and harder-to-reach groups. Note: The percentage of women who have both knowledge and intention, and who have neither knowledge nor intention, is shown in Supplementary Table 2, alongside the percentage who have knowledge and intention.

We examined two proxies of ability and willingness to use FP - knowledge of FP methods and source, and intention to use FP, respectively - among women with unmet need (Fig. 3, Supplementary material 2). Knowledge of FP increased over time in all groups: among easier-to-reach women with unmet need, it rose from 53% to 85% between 1997 and 2014. FP knowledge was similar among unmarried women (90% in 2014), and somewhat lower among the rural poor (76%). Adolescents with unmet need had much lower knowledge of FP, with only half knowing of pills and injectables as well as a FP source by 2012/13, rising to 60% in 2014.

Intention to use was consistently highest among unmarried women with unmet need, rising to 75% in 2014. In contrast, among easier-to-reach women, adolescents and the rural poor, intention to use FP rose between 1997 and 2005, and has since stagnated around 40%. Among women with unmet need, the proportion with neither knowledge of nor intention to use FP was lower than the proportion with both knowledge and intention by 2014, in all groups except for adolescents (Supplementary material 2).

### Determinants of FP knowledge and intention to use FP among women with unmet need

Among all women with unmet need in 2014, knowledge of FP increased with age, parity, education and wealth (Table 2). It was also higher among women living in urban areas, in the west, and among polygamous or formerly married women (compared with women in rural areas, in other zones, and never-married or monogamous currently married women, respectively). After adjusting for all socio-demographic characteristics, women with fewer living children, without formal education, and living outside of the western zone had lower odds of FP knowledge; the association of knowledge with age, marital status, location, and wealth disappeared.

Intention to use FP was higher among never-married women compared to monogamous married women, and lower in rural than urban areas, in the north, and among women with no education. Women with knowledge of FP were more than twice as likely to intend to use FP in future as women without knowledge (crude OR: 2.37, 95% CI: 1.61–3.50). There was no clear association between intention to use and woman’s age, parity, or wealth.

After adjusting for all characteristics, never-married women (adjusted OR: 3.04, 95% CI: 1.46–6.34) and women with FP knowledge (adjusted OR: 2.10, 95% CI: 1.36–3.24) were more likely to intend to use FP. Compared to the west, women in the north were less likely and women in the south more likely to intend to use. Intention to use also increased with number of living children, although urban and better educated women no longer had higher odds of intention to use.

## Discussion

This is the first in-depth analysis comparing FP trends over several decades among subgroups of women with different barriers to accessing FP in Senegal. Our findings show that the recent rapid acceleration in MCPR among WRAU was also observed among easier-to-reach and rural poor women, and to a lesser extent among adolescents and unmarried women. A slight decline in unmet need was observed among easier-to-reach women, but not among harder-to-reach groups. Substantial gains in percent demand satisfied were observed in all groups, however the percentage of met need remains low overall, and large differences persisted between groups in 2014: compared with WRAU (44%), percent demand satisfied was lower among adolescents (39%), and lowest among rural poor women (27%). Our findings further highlight that while knowledge of FP is relatively high among women with unmet need – with the notable exception of adolescents – fewer than half of them intend to use FP in future among all groups except for unmarried women.

### Trends in FP indicators

The higher percent demand satisfied observed among unmarried women is unexpected, since they are thought to experience more barriers to FP use than easier-to-reach women. Unmarried women were wealthier and more educated than women in other groups. However, these estimates are based on fewer than 100 unmarried women with unmet need in each survey, leading to wide uncertainty intervals. Moreover, sexual activity among unmarried women is likely to be underreported, due to the strong stigma against pre- and extra-marital sex in Senegal^31^,^32^. Unmarried women are only categorised as having an unmet need if they report sexual activity in the last month, therefore the unmet need for FP is likely to be underestimated in our study, and the percent demand satisfied overestimated, among unmarried women and adolescents. Nonetheless, the higher percent demand satisfied in this group may also partly reflect the fact that births out of wedlock are highly stigmatised in Senegal, increasing unmarried women’s motivation to use FP. Selection bias may also contribute to this trend, if unmarried women who are comfortable reporting sexual activity are also more empowered to access contraception despite existing barriers.

The much lower levels of percent demand satisfied among rural poor women highlight substantial barriers to access in this group, probably due to a constellation of obstacles including geographical and financial constraints, as well as opposition to FP use. Although in some countries contraception is provided free of charge, user charges for FP services and products are on average $3 in Senegal, where over one-third of the population lives below $1.90/day^33^,^34^. Moreover, approval of contraception is strongly associated with education^35^, and formal education remains low among women and men in rural areas.

Overall, percent demand satisfied remains low in Senegal: in 2014, fewer than half of easier-to-reach women and adolescents in need of FP – and fewer than 30% of rural poor women – were using a modern method. These findings echo high levels of unintended pregnancies found in urban Senegal, higher among poorer women^36^, and indicate that there are still substantial barriers to contraceptive use that need to be addressed. The stagnation in unmet need observed among harder-to-reach women does not necessarily indicate a lack of progress in these groups: in a context of rising percent demand satisfied, this lack of change rather reflects parallel increases in MCPR and in the total demand for FP (defined as the sum of contraceptive use and unmet need).

The considerable rise in percent demand satisfied from 2010 to present in groups facing greater barriers to FP access – as well as easier-to-reach women – is a substantial achievement for the Senegalese Ministry of Health and other stakeholders. Although the National FP Action Plan was implemented from 2012 onward, financial and resource investments gained momentum several years earlier after a renewed interest in FP internationally^37^. In Senegal, this was reflected by commitments to double the Ministry of Health Family Planning budget^8^ and increased investment by key external donors^11^,^37^,^38^. FP activities carried out by other actors, and secular trends in economic growth and urbanisation are likely to have contributed to this rise. Nonetheless, the clear departure from the pre-2010/11 trend suggests that increased FP investments contributed to the accelerated rise in contraceptive use in all subgroups. Senegal had one of the slowest increases in MCPR in the West African region between 1990 and 2010^39^, however current contraceptive use is substantially higher than in neighbouring francophone countries including Mali, Mauritania and Guinea (with similar levels of unmet need), but lower than in Ghana^2^.

Several factors have been argued to contribute to similar rapid FP achievements in two other countries – Ethiopia and Rwanda, with MCPRs of 29% and 52% respectively^2^. Among these, strong political leadership, substantial financial commitments from the Ministry of Health and development partners, and strategic partnerships with NGOs or private companies have also been observed in Senegal^40^,^41^,^42^. One key difference between Senegal and these countries, however, is that contraceptives are provided for free in Ethiopia and Rwanda. Another difference is their focus on community-based distribution of contraceptives by trained community health workers in village-level facilities, as well as through door-to-door distribution^40^,^43^. Although official guidelines permit community health agents in Senegal to administer reversible methods including implants, they do not perform door-to-door distribution and only 2% of users obtained their methods at the community level in 2014 (in health huts, community pharmacies, or outreach/mobile teams)^15^.

### Barriers to FP use among women with unmet need

We examined drivers of FP use within the “ready, willing, able” framework^27^,^28^, adapting an approach used by Machiyama and Cleland^29^. Knowledge of FP (one dimension of *ability*) increased to relatively high levels among easier-to-reach, unmarried and rural poor women with unmet need – although less than two-thirds of adolescents knew of FP methods and sources, suggesting that reliable FP information is not reaching younger women. However, intention to use FP (our proxy for *willingness*) has only increased slightly since 2005, and over half of women with unmet need do not intend to use FP in the future in all groups except for unmarried women, indicating that acceptability of FP is low throughout the majority of Senegalese population. These results confirm other reports from Senegal suggesting that opposition to contraceptive use remains widespread^16^,^17^, despite information and advocacy campaigns. Another DHS analysis found that, after not being at risk of conception (due to post-partum amenorrhea or infrequent sexual intercourse), the two most common reasons for not using contraception were opposition to contraceptive use by themselves or someone close to them, and concerns with contraceptive side-effects or health risks^31^. Health providers have a role to play in dispelling myths about FP, as well as in helping women find a better-suited method if side effects occur (including nausea, headaches, and menstrual irregularity associated with hormonal methods)^44^. However, two Service Provision Assessment analyses have found that the quality of FP counselling in Senegal is generally poor, with few providers counselling on side effects and follow-up services in particular^45^,^46^.

In our study, over one-third of women with unmet need had both knowledge of and intention to use FP; the fact that they were not using contraception indicates that other important barriers to use remain. Intention to use FP is probably a good proxy for *willingness*; although some women may be limited by opposition from their partner or other relatives^35^. In addition, it is likely that a significant proportion of women with knowledge and intention are not using FP because of *ability*-related barriers other than lack of knowledge, including geographical and financial barriers. In rural areas, 14% of people were located more than one hour travel time away from the closest facility, compared to 5% in Dakar^47^. Furthermore, only 87% of facilities nationally were found to offer FP services in 2014 (less in the South and West of the country)^48^. There is also evidence that all contraceptive methods were not always available in all facilities, particularly in Southern regions and in one Central region (Fatick)^48^,^49^ – although a national intervention aiming to strengthen the contraceptive supply chain had not been rolled out in the South at the time of the survey^49^ – and that providers place age or marital status restrictions on contraceptive provision^6^,^16^,^50^. Unlike other countries, FP services are not provided for free in Senegal. These findings suggest that access to facilities and access to FP services within facilities remain problematic in Senegal, contrary to what has been reported in Ghana^29^. In particular, it is important to understand what barriers unmarried women face, since over two-thirds of those with unmet need have both knowledge and intention.

### Strengths and limitations

A strength of our analysis was the use of three inter-related indicators of FP. While MCPR and unmet need for FP are routinely reported, the relevance of the percent demand satisfied for monitoring policies and programmes has recently been highlighted^21^, and it has been argued that since the denominator captures only women in need of FP, percent demand satisfied should be referred to as “family planning coverage”^51^. In contrast to the often-used wealth quintiles, we examined trends among four groups who are more easily identifiable to decision-makers, and thus can be used to recognise gaps in coverage and target strategies. Our analyses benefitted from the availability of standardised, nationally representative data on FP use and fertility intentions from the DHS, enabling us to compare FP coverage among subgroups of women as well as all WRAU.

Several limitations to DHS data are worth noting because of their potential impact on our findings. First, while the DHS questions are generally standardised, there are sometimes changes from one survey to the next requiring careful attention to standardise categories^52^; we re-coded all variables in the dataset to ensure comparability, as necessary. Changes in survey methodology may explain the apparent decline in MCPR among unmarried women between 1997 and 2005 (Fig. 1b), since policy changes are unlikely to have affected only this group. Second, in addition to the underreporting of sexual activity among unmarried women and adolescents, the assumption that married women are all exposed to sex may mean that the demand for FP is overestimated, and hence the percent demand satisfied is underestimated, in this group. Third, we were unable to examine financial or geographical access – two dimensions of ability – using DHS data.

### Policy and research implications

As the 2016–2018 National FP Action Plan begins, it is timely to review historical FP trends in Senegal to inform future strategies nationally, and in other West African countries. Following the important progress achieved in recent years, it is essential to build on efforts to improve access to FP for all women, and particularly for rural poor women and adolescents. Although the national target for MCPR was set among WRAU, our study highlights the importance of also tracking percent demand satisfied among groups known to experience more barriers to access as well as WRAU, in order to better target interventions and ensure progress is equitable. Together, adolescents, unmarried and rural poor women account for over 1.7 m women in Senegal, or 51% of women of reproductive age and at least one-quarter of women in need of FP. Our findings indicate that there is a need to address low knowledge of FP among adolescents, and low acceptability of FP throughout Senegal, especially in the North and in rural areas. Current outreach strategies targeting adolescents, including training of peer educators, communication campaigns, and school-based interventions^8^,^53^,^54^ need to be evaluated and scaled up if found to be effective. More research (particularly qualitative) is needed to understand reasons for low willingness to use FP among women with unmet need, such as the contribution of concerns with side-effects and opposition by partners, in order to design more effective communication campaigns.

Efforts should be made to reinforce access to FP services among all women, and for rural poor women and adolescents in particular. We echo recommendations by Sidze *et al*.^6^ for the right to access FP irrespective of age or marital status to be reaffirmed in policy documents and provider trainings, to help eliminate provider-imposed restrictions to contraception. Affordability of contraceptive services and transport need to be better understood in order to tailor insurance schemes or subsidies should harder-to-reach groups continue to face barriers accessing FP. Family planning was not included in the strategic plan for Universal Health Coverage, due to be launched in 2017 55; the cost-effectiveness of including contraception should be revisited. Other innovative strategies need to be scaled up to increase access: among these, the expansion of community-based distribution outlined in the National FP Action Plan seems promising^8^. Although door-to-door distribution may not be cost-effective in Senegal, the low percentage of users obtaining methods through health huts or outreach events point to a missed opportunity. Community health agents need to receive more in-depth FP training in order to engage women coming for non-FP visits, and help address misconceptions about FP. Lastly, FP should be integrated into all monthly outreach activities (“stratégies avancées”) in rural areas conducted by health posts, with the support of reproductive health partners, to ensure all women and men who wish to use contraception are able to do so.

## Additional Information

**How to cite this article:** Cavallaro, F. L. *et al*. Examining trends in family planning among harder-to-reach women in Senegal 1992–2014. *Sci. Rep.* **7**, 41006; doi: 10.1038/srep41006 (2017).

**Publisher's note:** Springer Nature remains neutral with regard to jurisdictional claims in published maps and institutional affiliations.

## Supplementary Material

Supplementary Information

## Figures and Tables

**Table 1:** Socio-demographic characteristics of women in easier-to-reach and harder-to-reach groups (2014).

Characteristics	Easier-to-reach	Adolescents	Unmarried	Rural poor
N	3,873	1,918	1,266	1,431
**Age group**				
15–19	0	100	0	0
20–29	39.1	0	69.3	48.0
30–39	38.0	0	18.9	32.5
40–49	22.9	0	11.8	19.5
**Number of living children**				
0	10.6	86.1	64.5	3.5
1	15.8	12.1	14.8	11.3
2–4	47.2	1.8	15.6	45.8
5 or more	26.5	0	5.1	39.4
**Marital status**				
Never married	0	76.8	71.3	0
Currently married - monogamous	66.9	20.3	0	61.9
Currently married - polygamous	33.1	2.6	0	38.1
Formerly married	0	0.4	28.7	0
**Type of residence (urban/rural)**				
Rural	41.3	49.5	23.9	100
Urban	58.7	50.5	76.1	0
**Zone**				
West	44.0	35.3	62.1	4
Centre	22.7	26.9	13.1	42.7
North	21.8	22.9	13.7	18.9
South	11.5	14.9	11.1	34.4
**Education**				
None	58.7	30.9	32.0	88.1
Primary	25.5	24.4	24.0	9.6
Secondary or higher	15.8	44.7	44.0	2.3
**Household wealth quintile**				
Poorest	1.1	17.9	5.6	100
Poorer	23.8	17.6	10.2	0
Middle	24.0	22.0	18.3	0
Richer	25.4	18.8	24.7	0
Richest	25.6	23.6	41.1	0

**Table 2:** Odds ratios for knowledge of FP and intention to use FP among women with unmet need (2014).

Socio-demographic characteristics	Knowledge of FP*			Intention to use FP**		
	N (% with knowledge)	Crude OR (95% CI)	Adjusted OR*** (95% CI)	N (% with intention)	Crude OR (95% CI)	Adjusted OR*** (95% CI)
**Age**						
15–19	150 (59.9)	0.40 (0.23–0.68)	0.89 (0.46–1.75)	150 (39.9)	0.92 (0.53–1.59)	1.64 (0.78–3.44)
20–29	652 (79.0)	1	1	652 (42.0)	1	1
30–39	543 (85.3)	1.53 (1.09–2.16)	1.28 (0.83–1.97)	543 (45.0)	1.13 (0.79–1.61)	0.91 (0.65–1.27)
40–49	300 (87.0)	1.77 (1.19–2.64)	1.37 (0.77–2.42)	300 (41.1)	0.96 (0.66–1.40)	0.71 (0.44–1.16)
**Number of living children**						
0	96 (55.1)	1	1	96 (34.9)	1	1
1	271 (77.2)	2.76 (1.33–5.74)	3.38 (1.66–6.89)	271 (30.2)	0.81 (0.42–1.54)	1.04 (0.54–2.01)
2–4	720 (84.6)	4.47 (2.32–8.60)	5.00 (2.26–11.04)	720 (48.0)	1.72 (0.88–3.36)	2.93 (1.31–6.54)
5 or more	558 (83.7)	4.19 (1.98–8.87)	4.85 (2.10–11.20)	558 (43.2)	1.42 (0.75–2.70)	3.58 (1.55–8.25)
**Marital status**						
Never married	56 (78.4)	0.95 (0.44–2.05)	1.12 (0.50–2.51)	56 (70.8)	3.04 (1.46–6.34)	3.53 (1.52–8.22)
Currently married - monogamous	1002 (79.2)	1	1	1002 (44.4)	1	1
Currently married - polygamous	568 (84.4)	1.42 (1.04–1.94)	1.28 (0.93–1.76)	568 (35.4)	0.69 (0.50–0.94)	0.59 (0.42–0.81)
Formerly married	19 (96.1)	6.51 (0.79–53.36)	3.81 (0.51–28.69)	19 (66.0)	2.44 (0.59–10.07)	1.62 (0.33–7.99)
**Type of residence**						
Rural	1147 (74.9)	0.33 (0.20–0.56)	0.63 (0.37–1.05)	1147 (36.3)	0.54 (0.37–0.80)	0.66 (0.43–1.01)
Urban	498 (89.9)	1	1	498 (51.2)	1	1
**Zone**						
West	189 (93.2)	1	1	189 (50.9)	1	1
Centre	527 (76.5)	0.24 (0.12–0.45)	0.39 (0.20–0.77)	527 (44.8)	0.78 (0.49–1.26)	1.54 (0.92–2.58)
North	444 (74.9)	0.22 (0.11–0.42)	0.38 (0.19–0.72)	444 (22.6)	0.28 (0.18–0.45)	0.51 (0.32–0.81)
South	485 (76.4)	0.24 (0.12–0.47)	0.38 (0.19–0.76)	485 (53.9)	1.13 (0.68–1.88)	2.13 (1.22–3.72)
**Education level**						
None	1168 (78.5)	0.57 (0.37–0.87)	0.59 (0.38–0.92)	1168 (39.2)	0.61 (0.44–0.87)	0.79 (0.57–1.10)
Primary	312 (86.5)	1	1	312 (51.2)	1	1
Secondary or higher	165 (87.6)	1.10 (0.56–2.16)	1.26 (0.58–2.74)	165 (47.4)	0.86 (0.52–1.41)	1.05 (0.51–2.13)
**Household wealth quintile**						
Poorest	529 (74.4)	0.23 (0.10–0.51)	0.71 (0.33–1.50)	529 (42.9)	0.80 (0.50–1.28)	0.83 (0.47–1.46)
Poorer	444 (74.6)	0.23 (0.11–0.51)	0.66 (0.34–1.30)	444 (34.2)	0.56 (0.34–0.91)	0.60 (0.35–1.02)
Middle	327 (79.7)	0.31 (0.16–0.62)	0.74 (0.40–1.36)	327 (38.0)	0.66 (0.41–1.06)	0.74 (0.43–1.28)
Richer	193 (86.5)	0.51 (0.22–1.18)	0.60 (0.27–1.31)	193 (50.8)	1.11 (0.69–1.78)	1.08 (0.61–1.92)
Richest	152 (92.7)	1	1	152 (48.3)	1	1
**FP knowledge**						
Does not have knowledge	—	—	—	360 (26.7)	1	1
Has knowledge	—	—	—	1285 (46.3)	2.37 (1.61–3.50)	2.10 (1.36–3.24)

^*^Knowledge of FP is defined as women knowing of pills and injectables, and of a source of FP commodities.

^**^Intention to use FP is defined as women reporting they plan to use FP in the future.

^***^Adjusted models include all socio-demographic characteristics as explanatory variable.
