# Supplementary material for: Examining trends in family planning among harder-to-reach women in Senegal 1992–2014
Source: Sci Rep. 2017 Jan 20;7:41006. doi: 10.1038/srep41006 (PMC5247687; doi:10.1038/srep41006)
Supplement: Supplementary Information [file srep41006-s1.doc]

Supplementary material 1. Change in modern contraceptive prevalence rate, unmet need and percent demand satisfied over time, among WRAU, and subgroups of women

|  | **N** | **MCPR*** | **95% CI** | **Absolute annual change from previous survey****** | **Unmet need for FP**** | **95% CI** | **Absolute annual change from previous survey****** | **Percent demand satisfied***** | **95% CI** | **Absolute annual change from previous survey****** |
| --- | --- | --- | --- | --- | --- | --- | --- | --- | --- | --- |
| **Women of reproductive age in union (national target)** | | | | | | | | | | |
| 1992/93 | 4,450 | 4.8 | 3.9-5.8 | 0.36 | 28.8 | 27.1-30.6 | - | 14.2 | 11.9-16.9 | - |
| 1997 | 6,030 | 8.1 | 6.7-9.8 | 0.79 | 35.0 | 33.4-36.6 | 1.48 | 18.8 | 15.9-22.2 | 1.10 |
| 2005 | 10,221 | 10.0 | 9.0-11.2 | 0.23 | 32.0 | 30.8-33.1 | -0.37 | 23.9 | 21.7-26.2 | 0.63 |
| 2010/11 | 10,804 | 11.9 | 10.8-13.1 | 0.33 | 30.1 | 28.7-31.5 | -0.33 | 28.4 | 26.1-30.9 | 0.78 |
| 2012/13 | 5,797 | 16.0 | 14.1-18.1 | 2.05 | 29.2 | 27.6-30.9 | -0.45 | 35.4 | 31.9-39.0 | 3.50 |
| 2014 | 5,820 | 20.2 | 18.0-22.5 | 3.00 | 25.6 | 23.8-27.4 | -2.57 | 44.1 | 40.3-48.0 | 6.21 |
| **Easier-to-reach women** | | | | | | | | | | |
| 1992/93 | - | - | - | - | - | - | - | - | - | - |
| 1997 | 4,222 | 10.7 | 8.9-12.8 | - | 35.5 | 33.7-37.3 | - | 23.2 | 19.8-27.1 | - |
| 2005 | 7,228 | 12.6 | 11.3-14.0 | 0.23 | 32.0 | 30.6-33.5 | -0.43 | 28.2 | 25.6-31.0 | 0.62 |
| 2010/11 | 7,239 | 14.5 | 13.0-16.0 | 0.33 | 29.7 | 28.0-31.5 | -0.40 | 32.7 | 29.9-35.7 | 0.78 |
| 2012/13 | 4,036 | 19.8 | 17.4-22.4 | 2.65 | 28.1 | 26.3-29.9 | -0.80 | 41.3 | 37.4-45.4 | 4.30 |
| 2014 | 3,873 | 23.0 | 20.3-25.9 | 2.29 | 25.0 | 22.9-27.2 | -2.21 | 47.9 | 43.6-52.2 | 4.71 |
| **Adolescents** | | | | | | | | | | |
| 1992/93 | 1,426 | 0.7 | 0.4-1.4 | 0.02 | 9.7 | 8.1-11.6 | - | 6.8 | 3.4-12.9 | - |
| 1997 | 1,958 | 1.2 | 0.8-1.9 | 0.12 | 10.9 | 9.4-12.6 | 0.29 | 10.2 | 6.8-15.0 | 0.81 |
| 2005 | 3,658 | 1.4 | 0.9-2.0 | 0.02 | 10.3 | 9.2-11.4 | -0.07 | 11.7 | 8.2-16.5 | 0.19 |
| 2010/11 | 3,604 | 1.9 | 1.3-2.8 | 0.09 | 9.1 | 7.8-10.5 | -0.21 | 17.3 | 12.1-24.1 | 0.97 |
| 2012/13 | 2,073 | 1.4 | 1.0-2.0 | -0.25 | 8.0 | 6.4-9.9 | -0.55 | 14.8 | 10.5-20.6 | -1.25 |
| 2014 | 1,918 | 4.1 | 2.3-7.1 | 1.93 | 6.3 | 4.8-8.1 | -1.21 | 39.4 | 25.6-55.1 | 17.57 |
| **Unmarried women >20** | | | | | | | | | | |
| 1992/93 | 844 | 7.7 | 6.0-9.8 | 0.14 | 6.6 | 5.0-8.8 | - | 53.7 | 44.5-62.7 | - |
| 1997 | 1,178 | 8.3 | 6.4-10.7 | 0.14 | 3.7 | 2.7-5.0 | -0.69 | 69.2 | 60.9-76.5 | 3.69 |
| 2005 | 1,862 | 4.1 | 2.9-5.8 | -0.52 | 2.7 | 1.9-3.8 | -0.12 | 60.2 | 47.7-71.5 | -1.11 |
| 2010/11 | 2,276 | 4.1 | 3.1-5.5 | 0.00 | 4.1 | 3.0-5.7 | 0.24 | 50.1 | 38.9-61.3 | -1.74 |
| 2012/13 | 1,285 | 4.8 | 3.6-6.5 | 0.35 | 3.2 | 2.2-4.5 | -0.45 | 60.4 | 50.1-69.9 | 5.15 |
| 2014 | 1,266 | 7.0 | 5.0-9.5 | 1.57 | 3.3 | 2.2-5.1 | 0.07 | 67.5 | 53.8-78.8 | 5.07 |
| **Rural poor women, married >20** | | | | | | | | | | |
| 1992/93 | - | - | - | - | - | - | - | - | - | - |
| 1997 | 1,235 | 0.9 | 0.5-1.8 | - | 34.7 | 31.8-37.7 | - | 2.6 | 1.3-5.1 | - |
| 2005 | 1,854 | 2.7 | 2.0-3.7 | 0.22 | 31.0 | 28.5-33.6 | -0.46 | 8.1 | 5.9-11.0 | 0.68 |
| 2010/11 | 2,569 | 4.4 | 3.5-5.6 | 0.29 | 31.1 | 28.9-33.3 | 0.02 | 12.4 | 9.9-15.4 | 0.74 |
| 2012/13 | 1,242 | 6.4 | 4.4-9.2 | 1.00 | 33.0 | 30.1-36.0 | 0.95 | 16.3 | 12.0-21.8 | 1.95 |
| 2014 | 1,431 | 11.3 | 9.1-13.9 | 3.50 | 30.1 | 27.6-32.7 | -2.07 | 27.3 | 22.5-32.6 | 7.86 |
|  |  |  |  |  |  |  |  |  |  |  |

*Modern contraceptive prevalence rate: percentage of all women using modern family planning methods

**Percentage of all women with unmet need for family planning

***Percentage of women in need of FP using modern family planning methods

****Absolute annual change was calculated as the difference in estimates between a survey and the previous one, divided by the number of years between the two surveys (calculated as years between the midpoint months of the two data collection periods).

Supplementary material 2: Knowledge of FP and intention to use FP over time among women with unmet need for FP, across subgroups of women

|  | 1997 | 2005 | 2010/11 | 2012/13 | 2014 |
| --- | --- | --- | --- | --- | --- |
| **All women with unmet need** |  |  |  |  |  |
| N | 2,168 | 3,304 | 3,389 | 1,804 | 1,645 |
| Have knowledge (total) | 45.7 | 65.9 | 69.6 | 74.1 | 81.3 |
| Have intention to use (total) | 9.3 | 38.7 | 40.1 | 42.1 | 42.7 |
| Have neither knowledge or intention to use | 50.5 | 26.4 | 22.8 | 19.3 | 13.7 |
| Have both knowledge and intention to use | 5.5 | 31.0 | 32.5 | 35.5 | 37.7 |
| **Easier-to-reach women** |  |  |  |  |  |
| N | 1,472 | 2,281 | 2,151 | 1,150 | 1,020 |
| Have knowledge (total) | 52.6 | 71.1 | 76.5 | 78.9 | 84.8 |
| Have intention to use (total) | 8.8 | 38.9 | 40.8 | 42.7 | 41.8 |
| Have neither knowledge or intention to use | 44.4 | 23.1 | 17.8 | 15.9 | 11.3 |
| Have both knowledge and intention to use | 5.8 | 33.1 | 35.1 | 37.5 | 37.9 |
| **Adolescents** |  |  |  |  |  |
| N | 214 | 403 | 360 | 185 | 150 |
| Have knowledge (total) | 33.9 | 43.0 | 48.0 | 47.1 | 59.9 |
| Have intention to use (total) | 11.8 | 33.9 | 35.5 | 29.4 | 39.9 |
| Have neither knowledge or intention to use | 60.9 | 43.9 | 40.0 | 42.6 | 29.2 |
| Have both knowledge and intention to use | 6.5 | 20.8 | 23.6 | 19.1 | 28.9 |
| **Unmarried women >20** |  |  |  |  |  |
| N | 51 | 49 | 100 | 56 | 47 |
| Have knowledge (total) | 47.2 | 65.0 | 74.0 | 76.3 | 89.5 |
| Have intention to use (total) | 22.1 | 57.7 | 49.2 | 64.4 | 75.2 |
| Have neither knowledge or intention to use | 48.4 | 11.9 | 14.5 | 16.7 | 3.4 |
| Have both knowledge and intention to use | 17.8 | 34.7 | 37.7 | 57.4 | 68.1 |
| **Rural poor women, married >20** |  |  |  |  |  |
| N | 431 | 571 | 778 | 413 | 428 |
| Have knowledge (total) | 23.7 | 59.9 | 52.7 | 69.9 | 75.5 |
| Have intention to use (total) | 8.2 | 38.7 | 37.9 | 43.3 | 40.6 |
| Have neither knowledge or intention to use | 70.3 | 29.9 | 35.3 | 20.4 | 18.5 |
| Have both knowledge and intention to use | 2.1 | 28.5 | 25.9 | 33.6 | 34.7 |
